# Clinical cell therapy imaging using a perfluorocarbon tracer and fluorine-19 MRI

**DOI:** 10.1002/mrm.25454

**Published:** 2014-09-19

**Authors:** Eric T Ahrens, Brooke M Helfer, Charles F O'Hanlon, Claudiu Schirda

**Affiliations:** 1 Department of Radiology, University of California at San DiegoLa Jolla, California, USA; 2 Department of Research and Development, Celsense, Inc.Pittsburgh, Pennsylvania, USA; 3 Department of Radiology, University of PittsburghPittsburgh, Pennsylvania, USA

**Keywords:** MRI, dendritic cells, cell tracking, fluorine-19, ^19^F, perfluorcarbon, immunotherapy, cancer

## Abstract

**Purpose:**

Cellular therapeutics are emerging as a treatment option for a host of serious human diseases. To accelerate clinical translation, noninvasive imaging of cell grafts in clinical trials can potentially be used to assess the initial delivery and behavior of cells.

**Methods:**

The use of a perfluorocarbon (PFC) tracer agent for clinical fluorine-19 (^19^F) MRI cell detection is described. This technology was used to detect immunotherapeutic dendritic cells (DCs) delivered to colorectal adenocarcinoma patients. Autologous DC vaccines were labeled with a PFC MRI agent ex vivo. Patients received DCs intradermally, and ^19^F spin-density-weighted MRI at 3 Tesla (T) was used to observe cells.

**Results:**

Spin-density-weighted ^19^F images at the injection site displayed DCs as background-free “hot-spot” images. ^19^F images were acquired in clinically relevant scan times (<10 min). Apparent DC numbers could be quantified in two patients from the ^19^F hot-spots and were observed to decrease by ∼50% at injection site by 24 h. From 3T phantom studies, the sensitivity limit for DC detection is estimated to be on the order of ∼10^5^ cells/voxel in this study.

**Conclusion:**

These results help to establish a clinically applicable means to track a broad range of cell types used in cell therapy. Magn Reson Med 72:1696–1701, 2014. © 2014 The Authors. Magnetic Resonance in Medicine Published by Wiley Periodicals, Inc. on behalf of International Society of Medicine in Resonance.

## INTRODUCTION

In vivo imaging can potentially aid in the clinical translation of emerging cell therapies by assessing the behavior of cells following transfer to the patient. Feedback regarding crucial determinants of the success of cell therapy, including the persistence, mobility, and optimal route of cell delivery, can be obtained repeatedly with use of an appropriately designed noninvasive imaging technology 1. Moreover, emerging cell therapies, such as those using engineered immune cells 2 and stem cells, can be slow to gain regulatory approval, in part, because clinical researchers are challenged to verify cellular locations and migration patterns over time.

MRI is emerging as an option for in vivo cell tracking 1. Prior clinical MRI cell tracking studies 3 have used clinically approved metal-ion based vascular contrast agents, used off-label, to tag cells ex vivo before transfer. However, these agents are not designed for intracellular labeling and often require transfection procedures to label nonphagocytic cells. Furthermore, the metal-ion based agents are detected indirectly by means of signal intensity (i.e., T_1_ or T_2_*) changes in proton anatomical images, making region of interest (ROI) quantification of grafted cell numbers difficult. Alternatives to MRI include radionuclide-based methods, however, these approaches are often of limited use for longitudinal studies because of finite radioisotope half-lives, as well as radiotoxicity concerns. Moreover, radionuclide-based images are unable to provide anatomical detail and are often combined with MRI or computed tomography images.

This study describes the first clinical experience using a perfluorocarbon (PFC) tracer agent specifically engineered for fluorine-19 (^19^F) MRI cell detection. Cells are labeled in culture using a PFC nanoemulsion formulation that is taken up by cells regardless of their phagocytic properties 4. Following transfer to the subject, cells are detected in vivo using ^19^F MRI 5. The fluorine inside the cells yields positive-signal “hot-spot” images, with no background signal due to the paucity of detectable fluorine atoms in host tissues. Images can be quantified to measure apparent cell numbers at sites of accumulation 5,6, thereby enabling “in vivo cytometry” 7. We describe initial cell detection results of a Phase I clinical trial for stage-4 colorectal cancer (CRC) treatment with an immunotherapeutic dendritic cell (DC) vaccine, where MRI was used to visualize cells after administration. Prepared DCs injected directly into peripheral tissue can potentially enter into the lymphatic system and lymph nodes and stimulate an anti-tumor, T cell response 8. The primary outcome measures of this trial 9 are (i) to observe any adverse events from the labeled DC vaccine and (ii) to investigate the ability to track labeled DCs by MRI; topic (ii) is described herein.

## METHODS

### Clinical Trial

This feasibility study was conducted under protocols approved by the University of Pittsburgh Cancer Institute Institutional Review Board and the Office of Cell, Tissue and Gene Therapy at the US Food and Drug Administration (BB-IND 14,730). A Drug Master File covering the commercially available PFC MRI tracer reagent (BB-MF 14,062) was cross-referenced in the IND application. The study 9 enrolled adult patients (N = 5 completed) with metastatic (stage 4) colorectal cancer. The patient study consisted of three separate intradermal administrations of a DC vaccine administered once per day for 3 days, where one of the doses was labeled with PFC. The number of labeled cells injected was initially 1 × 10^6^ DCs (N = 2, low or safety dose) and then increased to 1 × 10^7^ cells (N = 3). Cells were inoculated unilaterally in right quadriceps near the inguinal crease.

### DC Preparation and PFC Labeling

The autologous live DC vaccine was prepared using a 7-day culture protocol, as previously described 10. After surgical tumor resection, the tumor cells were mechanically minced, rendered apoptotic using ultraviolet B and γ radiation, lysed using collagenase and frozen. At least 2 weeks following surgery, patients underwent leukapheresis, and mononuclear cells were isolated by the Elutra System (CardianBCT, Inc., Lakewood, CO) and plated at 1.5 × 10^6^ cells/mL. The monocytes were separated into multiple lots. All cell lots were incubated with Genix DC Media (Cell Genix, Inc., Freiburg, Germany), GM-CSF (1000 U/mL, Berlix, Inc., Seattle, WA) and interlukin-4 (1,000 U/mL, Cell Genix) for 5 days. On day 6, the cells were additionally incubated with an activating cocktail 10 of interferon α (3000 U/mL, Schering-Plough, Kenilworth NJ), interferon γ (1000 U/mL, Actimmune, Inc., Brisbane, CA), interleukin 1β (25 ng/mL, Cell Genix), tumor necrosis factor α (5 ng/mL, Cell Genix), poly IC (20 µg/mL, InvivoGen, Inc., San Diego, CA), Immucothel (50 µg/mL, Biosyn, Inc., Carlsbad, CA), and thawed tumor lysate, as prepared above. Also, on day 6, clinical-grade PFC agent (CS-1000, Celsense, Inc., Pittsburgh, PA) at 2.5 mg/mL was added to a portion of cells. On day 7, all lots were washed three times, the cells counted, and aliquots removed for testing and quality assurance. Cell product release criteria included thresholds for bacterial contamination, the presence of endotoxins, and cell viability. Nuclear magnetic resonance (NMR) was used to assay the ^19^F content of the DCs after PFC labeling 4,6. Briefly, cell pellets containing a known cell number (∼3 × 10^6^) were lysed with 1% Triton X-100 (Arcos Organics), and 0.1% (v/v) trifluoroacetic acid (TFA, Sigma-Aldrich, St Louis, MO) was added as an NMR reference compound. The mixture was placed in an NMR tube, and one-dimensional (1D) ^19^F spectra were acquired at 470 MHz using a high resolution spectrometer (Bruker, Inc., Billerica, MA). The ^19^F spectra display two narrow major peaks, one each for PFC and TFA, with a chemical shift difference of −15.58 ppm. The ratio of the integrated areas under these two peaks was used to calculate the mean ^19^F/cell, as described previously 4,6. Cells were assayed for viability, using a standard trypan blue exclusion method. Four-color flow cytometry (Epics XL, Beckmann Coulter, Brea, CA) was performed by the University of Pittsburgh Cancer Institute Cell Processing Laboratory using fluorochrome-conjugated antibodies to measure expression levels of HLA-DR, CD83, CD86, and CCR7 to compare PFC-labeled versus unlabeled cells. HLA-DR is expressed on antigen presenting cells. CD83 and CD86 are markers for DC maturation. CCR7 is a marker for DC motility.

### In Vivo MRI

At 4 and 24 h after DC inoculation, subjects had ^19^F/^1^H MRI scans using a 3 Tesla (T) instrument (Tim Trio, Siemens, Inc., Erlangen, Germany). A custom-built ^19^F/^1^H 7 cm diameter transmit/receive surface coil (Stark Contrast, Inc., Erlangen, Germany) in a plastic housing was centered directly over the injection site and taped against the patient. A mechanical switch on the coil housing toggled between ^19^F and ^1^H; to switch nuclei between serial ^19^F/^1^H scans, the subject was removed and reinserted into the scanner while lying still on the patient bed. A ^19^F reference tube, consisting of 0.250 mL of 0.50% by volume of TFA was affixed to the center of the coil housing. The TFA chemical shift with respect to the PFC causes an ∼11.9 pixel registration displacement of the TFA tube that can be corrected ad hoc with postprocessing. The TFA sample was doped with 1.0 mM MnCl (Sigma-Aldrich, St. Louis, MO) to accelerate the T_1_ relaxation rate to match the PFC compound. The CS-1000 agent has T_1_/T_2_ = 470/250 ms at 3T and at 37°C. The ^19^F images were acquired using Siemens' standard spin-density weighted Fast Low Angle SHot (FLASH) sequence with a 9.5 min total scan time; the imaging parameters were repetition time/echo time (TR/TE) = 100/4.15 ms, NA = 96, flip angle (FA) = 45°, slice thickness 2 cm, number of slices (NS) = 3, field of view (FOV) = (28.8 cm)^2^, and matrix size 64 × 64. For anatomical reference, coregistered ^1^H FLASH images were collected with parameters TR/TE = 115/4.92 ms, NA = 2, FA = 25°, slice thickness 5 mm, NS = 12, FOV = (28.8 cm)^2^, and matrix size 192 × 192. A nonselective 1-pulse ^19^F MRS sequence was also used, with pulse width 0.5 ms, repetition/echo time (TR/TE) = 1500/0.35 ms, spectral width = 10 kHz, number of averages (NA) = 384.

Composite ^19^F/^1^H overlay images were created; the ^19^F images were manually threshold for visualization purposes to mask noise, and rendered in “hot-iron” pseudocolor scale using ImageJ software, while the ^1^H was in grayscale. The ^19^F image intensity profile from the inhomogeneous surface coil was not corrected, as the cell injection site was superficial and in close proximity to the TFA reference.

The number of apparent cells in hotspot voxels were computed directly from the in vivo images using methods previously described 4,6,7. In brief, the mean noise intensity for an image, (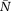), was calculated using voxels on the image border devoid of ^19^F signal. The signal to noise ratio (SNR) was calculated for each voxel in the image using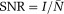, where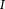 represents the voxel's intensity. A threshold mask of SNR = 2.5 was then applied to the images, which removes 99.3% of voxels containing only noise. Magnitude images have a non-zero mean pixel value in regions of noise, which can introduce a noise-dependent bias having a Rician distribution 11 that is nonnegligible when the SNR is low. All remaining voxels had their intensity corrected using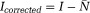. The total number of nuclei per voxel, and the summation for ROI, in cell hotspots was computed using the signal from the known number of nuclei in the TFA reference and using the per-patient cell labeling efficiency measured by means of NMR (see above) 6.

### Phantom MRI Studies

To benchmark the 3T MRI scanner sensitivity to ^19^F-labeled DCs, a cell phantom was constructed. Nonpatient human DCs were prepared from commercially obtained buffy coats (Central Blood Bank, Pittsburgh, PA) and labeled with PFC at an average of 3.74 × 10^12 19^F/cell, as described previously 4. A compacted pellet (1 × 10^7^ cells) of fixed cells was formed at the bottom of a conical tube by centrifugation, the supernatant removed, and the remaining tube volume was filled with 2% agarose doped with 30 mM NaCl. The sealed conical tube was imbedded into a 2 L vessel containing 2% agarose and salt, with the DC pellet positioned approximately 2 cm below the agarose surface. The ^19^F/^1^H surface coil was centered over the cell pellet, and ^19^F/^1^H MRI data were acquired in the scanner using the same parameters as the in vivo data. Resulting image data were visualized and analyzed using the above methods.

## RESULTS

**Figure 1:**
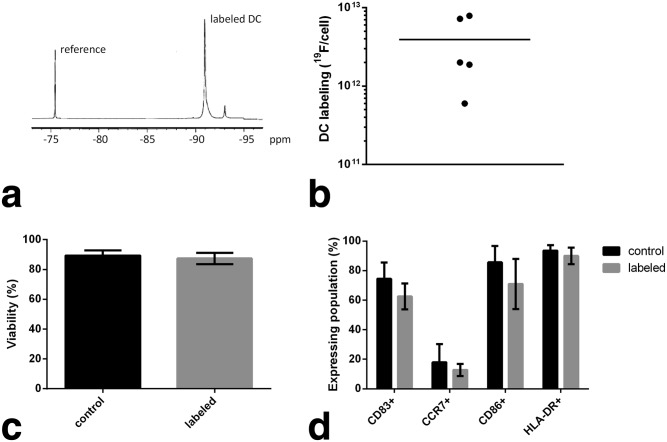
Patient DCs are efficiently labeled with PFC in culture without changes in viability or phenotype. a: A ^19^F NMR spectrum of a patient's DCs labeled with PFC; these spectra are used to measure the amount of ^19^F taken up by cells after labeling in culture. The PFC spectrum displays a major peak at approximately −91 ppm, and a small minor peak at −93 ppm that is generally undetectable in vivo. The NMR sample was doped with a reference compound (TFA) at −76 ppm. The relative area under the PFC and TFA peaks is used to calculate the cell labeling efficiency. b: A summary of ^19^F quantification results in DCs in the patient cohort. The horizontal bar is the average labeling for population (3.9×10^12 19^F/cell). c: DC viability, assayed using trypan blue exclusion, for PFC labeled and mock-labeled (control) patient DCs. d: Cell surface marker expression levels of key DC phenotypic markers, measured using flow cytometry. Labeled cells versus control DCs display no significant phenotypic differences.

We show that the clinical PFC agent safely labels patient cells ex vivo. Autologous patient mononuclear cells, obtained by means of leukapheresis, were differentiated into a DC vaccine using a 7-day culture regimen 10 that was modified to include PFC cell labeling (see Methods). After labeling, NMR analyses (Fig. 1a) revealed that patients' DCs contained an average number of fluorine atoms ranging from approximately 10^12^ to 10^13 19^F/cell (Fig. 1b), depending on batch, with a mean value (N = 5) of 3.9 × 10^12 19^F/cell (standard deviation 3.4 × 10^12^); this labeling efficiency is comparable to normal donor DC preparations 4. The labeled cell viability remained >95% compared with unlabeled DCs, as assessed by trypan blue exclusion assay (Fig. 1c). No statistically significant changes (by means of 2-way analysis of variance with Sidak's multiple comparisons and alpha = 0.05) in expression levels of HLA-DR, CD83, CD86, and CCR7 were observed by flow cytometry (Fig. 1d).

**Figure 2:**
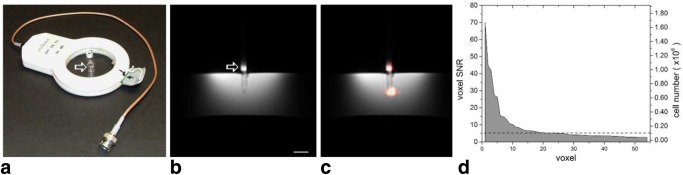
Clinical hardware modifications and benchmark of cell sensitivity by means of DC phantom studies. a: A custom-built ^19^F/^1^H surface coil add-on for a clinical 3T scanner with integrated external reference tube containing TFA (arrow). b: ^1^H phantom images of PFC labeled cell pellet (1 × 10^7^ cells) in conical tube and embedded in agarose filled vessel. Scale bar = 1 cm. c: Composite ^19^F/^1^H image with the ^19^F (DCs) rendered in hot-iron scale and the ^1^H in grayscale. Images were acquired using the same 3T MRI scanner configuration, pulse sequences, and scan times as the patients. The ^19^F image is threshold manually for display purposes. d: ^19^F MRI quantification results of the voxel-wise SNR and apparent cell number. A SNR = 2.5 cutoff was applied to the data. The dashed line is positioned at 10^5^ cells per voxel, the order of magnitude for cell detection sensitivity using current methods, as previously reported 5.

Initially, we conducted phantom studies to investigate the detection sensitivity in the same clinical MRI scanner that was used for patients in the trial. A phantom consisting of a PFC-labeled human DC pellet (1 × 10^7^ cells, labeled at 3.7 × 10^12 19^F/cell) was embedded in agarose at ∼2 cm below the phantom surface; images (Fig. 2) were acquired using the same 3T MRI scanner configuration, customized surface coil and reference (Fig. 2a), pulse sequences, and scan times as was used with patients. Figures 2b–c show resulting phantom images, and as expected, the signal from the cell pellet (hot-iron, pseudocolor) is localized and intense. Figure 2d shows quantification results from the ^19^F image and displays the voxel-wise SNR and proportionate cell number on the left and right axes, respectively. In Figure 2d, only voxels with SNR > 2.5 are displayed. We note that the isolated pool of signal from the cell pellet had a sizable point spread function that leaked signal beyond the boundary of the conical tube containing the cell pellet; quantification results from these external voxels are present in Figure 2d, as they contain signal from the cell pellet that is above the SNR = 2.5 threshold. The dashed line in Figure 2d at 10^5^ cells/voxel is the previously estimated 5 order of magnitude cell detection limit in a clinical scanner; these data show that this a conservative estimate using our experiment configuration. Importantly, the ^19^F image is only required to display localized “pools” of cells at low SNR, and the ^1^H images provide detailed anatomical context. The cell type labeled (i.e., larger cells can be labeled with more agent per cell), image acquisition methods and magnetic field strength, as well as radiofrequency coil configuration and placement, determine the actual sensitivity achievable for a particular study 12.

**Figure 3:**
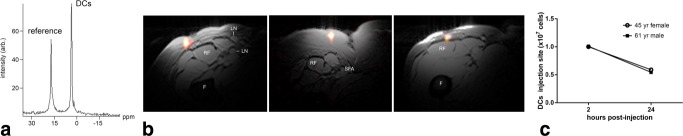
In vivo MRI in patients following intradermal DC administration into quadriceps. In these patients, approximately 1 × 10^7^ labeled cells were injected. a: shows a representative ^19^F MRS spectrum of patient at 4 h postinoculation. The DCs appear as a single narrow peak. “Reference” is from an external tube containing TFA placed alongside the patient. b: Axial composite ^19^F/^1^H images of the right thigh at 4 h postinoculation in three patients, a 53-year-old female (left), a 45-year-old female (middle) and a 61-year-old male (right), where the DCs are rendered in “hot-iron” pseudocolor and the ^1^H anatomy is displayed in grayscale (F = femur, RF = rectus femoris, SFA = superficial femoral artery, LN = inguinal lymph node). c: The results of the in vivo quantification of apparent cell numbers using the ^19^F MRI data, measured in two patients. By ∼24 h postinoculation, roughly half of the injected DCs were still present at the injection site.

We show that ^19^F-based MRI cell detection is feasible in patients using a clinical scanner within acceptable scan times. Figure 3 shows patient results. The representative in vivo ^19^F MRS (Fig. 3a) displays two peaks, a single peak from PFC in labeled DCs, and one from the external ^19^F reference solution (TFA) in a tube placed next to the quadriceps. Figure 3b displays composite ^19^F/^1^H MRI images from three different patients receiving the higher cell doses (1 × 10^7^). Signal from the injected cells (hot-iron, pseudocolor) is localized and intense. The ^19^F signal was contained within a single image slice (2 cm thick) for all but one patient. The TFA reference is out of plane. The total scan time for the ^19^F imaging was 9.5 min. The coregistered ^1^H MRI scan (Fig. 3b) provides a grayscale underlay and is annotated for anatomical reference.

Fluorine-based cell detection enables cell quantification directly from the in vivo images 6. We quantified the number of DCs within ^19^F image hot-spots in two patients where we were able to get complete datasets (45 year female and 61 year male). At ∼4 h posttransfer, we observed no significant change in the number of cells that had been injected (Fig. 3c). However, by ∼24 h posttransfer, DC numbers decreased to approximately half of the original values (Fig. 3c). This observation is consistent with cell efflux from the initial injection site as a result of cell migration to nearby lymph nodes or other tissues, or due to cell death and subsequent clearance of PFC material. We note that inguinal lymph nodes were captured within the MRI field of view, but there was no MRI evidence of cell accumulation in these tissues. Cell hotspots in Figure 3b had (maximum voxel SNR, mean voxel SNR, number of voxels with SNR > 2.5) equal to (39.4, 8.7, 24.0) and (51.3, 8.8, 16) for the 45-year-old female and 61-year-old male, respectively. In one of the subjects, 53-year-old female, presumed patient movement during the scan hindered our ability to quantify cells; subject motion causes ^19^F signal loss and can hinder accurate spin quantification 13.

The ^19^F MRI signals from two patients receiving lower (safety) doses (1 × 10^6^) were not reliably observed (data not shown). If one assumes that the cell dose disperses within a comparable tissue volume as the higher dose patients, we speculate that the low dose patients would have a cell density on the order of, or below, the cell detection limit threshold for our experimental configuration. Also, we note that in one of these patients the PFC cell labeling efficiency was very low (lowest point, Figure 1b) which further limits detectability.

## DISCUSSION AND CONCLUSIONS

We describe the use of a PFC tracer agent for detection of immunotherapeutic DCs delivered to colorectal adenocarcinoma patients. The PFC-based cell labeling agent was designed and optimized specifically for clinical MRI. Prior clinical MRI cell tracking work (e.g., de Vries et al) 14 has relied on off-label use of metal-ion-based nanoparticles, often in conjunction with transfection agents 15. Historically, various PFC molecules have been contemplated for clinical use as artificial oxygen carriers 16 in large doses (∼10 g/kg). For MRI cell imaging applications, a relatively miniscule quantity of PFC (∼80 µg/kg), contained within the transferred cells, is delivered to the subject. The PFC agent used (CS-1000 see the Methods section) was rigorously tested for acute toxicity in vivo, cytotoxicity in a range of cell types, and genotoxicity. In acute toxicity preclinical studies, no adverse events were noted at doses (per body weight) of order ∼100 times greater than anticipated in cellular therapy trials. The PFC formulation uses a novel perfluoropolyether molecule that has essentially one major NMR peak (Figs. 1a, 2a) and a relatively short T1 relaxation time (see the Methods section). Moreover, the PFC is formulated for use without transfection agents to gain entry into nonphagocytic cells 4. Only viable cells are labeled in culture by endocytic processes. The cell remains labeled as long as it remains viable, the PFC is not degraded once inside the cell, and there is no evidence for active exocytosis of the PFC label (unpublished observations).

An innovative aspect of the regulatory path used in the translation of this PFC agent is the use of a United States Food and Drug Administration (FDA) Drug Master File (DMF). The DMF generally contains detailed manufacturing information, as well as a compendium of regulation-required toxicity studies, and is typically used to disclose manufacturing conditions and safety data for therapeutic excipients (e.g., bulking or colorization agents). Because the amount of PFC delivered to the body is so small, the safety concerns mostly center on how the PFC labeling may alter the phenotype and function of the cells and whether labeling diminishes any putative therapeutic capacity of the cell. Our in vitro results (Fig. 1), and those reported elsewhere 4, support the view that this reagent is safe for cells and does not modify their therapeutic capacity. Importantly, the DMF can be cross-referenced in multiple Investigative New Drug applications submitted to the FDA, thus enabling integration of this imaging technology into a broad array of cell therapy products.

Although the mature DCs used in this study do not divide, generally, with PFC labeled cells having a mitotic phenotype, cell division and subsequent dilution of the intracellular label can potentially limit long-term studies of itinerant cells and decrease the accuracy of cell quantification. Death of labeled cells can lead to dispersion of the reagent and loss of ^19^F signal. Potentially, the PFC droplets can also be transferred to macrophages that have engulfed dead cells. If a large number of macrophages remain in an ROI, false positive signals could result. These caveats are the same for many commonly used imaging modalities where a tracer material is used, such as Indium-111 SPECT probes and various nanoparticle probes (e.g., iron-oxide).

Clinical scanners are generally designed to be specialized for ^1^H-only applications, but most can be adapted to scan ^19^F with the addition of a ^19^F/^1^H coil. However, some scanner manufacturers will also require additional hardware modifications to enable multinuclear data acquisitions 12. This initial clinical study used rudimentary data acquisition methods and a suboptimal MRI detector (surface coil) design, which had a limited and nonuniform cell detection profile. Importantly, implementation of accelerated MRI data acquisition methods 17 and advanced detectors (e.g., multiarray coils) 18 will certainly extend the sensitivity and utility of this technology. Additionally, consistent and accurate ^19^F spin quantification could benefit from known motion correction methods 13, but these were beyond the scope of this study.

Imaging the initial cell location and behavior over time in vivo provides critical feedback about cell delivery success on a per-patient basis, and may potentially provide a surrogate indicator of therapeutic outcomes. Failure to observe a clinical response raises the question as to whether a sufficient number of cells were delivered to, and/or persisted at the desired site(s). Conversely, the manifestation of undesired side effects raises the question as to whether a significant number of cells accumulated off-target. The ability to noninvasively quantify cellular behavior in human clinical trials will significantly enhance our ability to ensure adequate safety surveillance. Moreover, imaging human cells in their native environment may help in the development of novel therapeutics for diseases where small and large animals may fail to accurately model the complexity of human disease. Overall, these results are a key milestone in establishing a clinically applicable means to track a broad range of cell types used in the emerging field of cell therapy.
